# Cross-tissue single-cell atlas reveals Id2 promotes sepsis-induced tissue damage by mediating macrophage metabolic reprogramming via Pkm2

**DOI:** 10.7150/ijbs.136855

**Published:** 2026-08-24

**Authors:** Xiaodan Wang, Yiyan Liu, Yuanqun Zhou, Liyong Zou, Xiaowei Zhou, Zhongyuan Du, Daiqin Bao, Yibo Zhao, Yu Zhu, Yue Wu, Xingnan Ouyang, Li Wang, Liangming Liu, Tao Li

**Affiliations:** 1 Department of Shock and Transfusion, Daping Hospital, Army Medical University, Chongqing, 400042, China.; 2 Department of Anesthesiology, Daping Hospital, Army Medical University, Chongqing, 400042, China.; 3 College of Pharmacy and Biological Engineering, Chongqing University of Technology, Chongqing, 400054, China.

**Keywords:** macrophages, sepsis, metabolic reprogramming, *Id2*, *Pkm2*

## Abstract

As important immune cells, macrophage polarization is directly related to tissue damage in sepsis, and the polarization of macrophages is associated with their metabolic patterns. Previous studies exploring macrophage function in sepsis mainly focused on specific tissues, lacking comprehensive comparisons between tissues. Herein, we performed single-cell RNA sequencing (scRNA-seq) to systematically profile macrophages derived from the brain, heart, intestine, lung, spleen, and peripheral blood mononuclear cells (PBMCs) under homeostatic and septic conditions. Under steady-state, we detected the markers of macrophages in different tissues, classified the macrophages into 10 functional subtypes and compared the differences in their distribution among tissues. In sepsis, we found that *Pkm2* and *Id2* played important roles in macrophage glycolysis. Mechanistically, *Id2* mediated the metabolic reprogramming of macrophages by regulating the chromatin accessibility of *Pkm2*. Helichrysetin, an inhibitor of *Id2*, could significantly alleviate tissue damage and increase the survival of septic mice. In summary, our research depicted a cross-tissue macrophage landscape at the single-cell level that encompasses both homeostasis and sepsis. We also provided a new target and a potential drug for the treatment of sepsis by inhibiting macrophage metabolic reprogramming.

## Introduction

Sepsis is a systemic inflammatory reaction caused by infection, characterized by host immune dysfunction. It is common in patients with serious diseases, especially those with severe trauma or infectious diseases^1^. According to the statistical report of World Health Organization (WHO), approximately 49 million people worldwide develop sepsis annually, with the death toll reaching 11 million, accounting for 20% of all global deaths^2^. As a core member of the innate immunity, macrophages play a significant role in sepsis. Macrophages dysfunction can lead to multi-tissue damage and circulatory disorders^3^-^5^. However, there is a lack of systematic comparison regarding the differences in response level, phenotype, and molecular mechanisms of macrophages across different tissues during sepsis. The comparative map of function changes in multi-tissue macrophages after sepsis has not yet been revealed.

Macrophages are important members of the immune system, playing a crucial role in the homeostasis of tissues. According to their different origins, tissue macrophages can be divided into resident macrophages and exogenous bone marrow-derived monocytes^6^. Tissue-resident macrophages are derived from erythro-myeloid progenitors originating in the yolk sac and have tissue specificity, which play an important role in embryonic development and tissue homeostasis^7^-^9^. Exogenous monocytes originate from hematopoietic stem cells in the bone marrow and could replenish tissue-resident macrophages following injury, infection and inflammation^6^, ^10^. The differences and similarities of resident macrophages across tissues under steady-state, the differences in the infiltration of exogenous monocytes into various tissues after sepsis, and the phenotypic changes in both exogenous monocytes and resident macrophages all remain to be explored in order to enhance our understanding of sepsis-induced multi-tissue injury.

In sepsis, macrophages can play a pro-inflammatory or anti-inflammatory role by changing their polarization state, participate in the disease process. Studies reveal that the polarization state and function of macrophages are related to their metabolism. Pro-inflammatory macrophages are characterized by enhanced glycolysis and pentose phosphate pathways. While anti-inflammatory macrophages rely more on oxidative phosphorylation and fatty acid oxidation to support their anti-inflammatory and tissue repair functions^11^. Macrophages in the inflammatory outbreak period of sepsis support the production of their pro-inflammatory cytokines through glycolysis^12^, ^13^. Given the close correlation between metabolic reprogramming and macrophage functional polarization in sepsis, we hypothesized that key regulatory factors modulating macrophage metabolism could serve as critical mediators of sepsis progression and viable therapeutic targets for sepsis treatment. In this study, we drew a functional transformation map of multi-tissue macrophages through scRNA-seq and identify *Id2* as a key factor that regulate metabolic reprogramming of macrophages in sepsis. We then treated the septic mice with the *Id2* inhibitor helichrysetin to observe its therapeutic effects on sepsis-induced multi-tissue damage. In summary, our study aims to provide a new therapeutic target and a drug with translational potential for sepsis treatment from the perspective of inhibiting metabolic reprogramming.

## Materials and Methods

### Septic mice model

We used cecal ligation and puncture (CLP) to construct the septic mice model^14^. 8-week-old male and female C57BL/6 mice (equal sex ratio) were anesthetized with 30mg/kg pentobarbital sodium. Opening in the center of the abdomen, exposing the cecum, ligating at a distance of 0.5 cm from the tail end of the cecum, puncturing the tail end with a 21G needle to extrude a tiny amount of feces, and then returning the cecum to the abdominal cavity, close the incision. Postoperatively, subcutaneous injection of 37 °C normal saline (50 ml/kg weight) was given for fluid resuscitation. Mice were fasted with free access to water and housed in a thermos statted cage maintained at 30 °C for close monitoring. Subsequent experimental observations were conducted 12 hours after surgery. The sham surgery group only performed open surgery and suturing operations, without ligation or puncture. For each tissue and each treatment condition, three mice were used to construct scRNA-seq libraries.

### Analysis of 72-hour survival rate of mice

Mice were intraperitoneally injected with 10mg/kg helichrysetin (dissolved in DMSO) or DMSO before CLP surgery. The mice were put back into their cages, singly housed for 72-h monitoring. Mortality was defined by no heartbeat or absence of respiration, and mice that survived over 72 h were euthanized by overdose of sodium pentobarbital (n=16 in each group). The survival time and 72 h survival rate of mice were individually analyzed by Kaplan-Meier survival analyses, and the log rank test.

### Flow cytometry

Single cells of heart were sorted from mice after PBS perfusion and enzymatic digestion in tissue dissociation solution (Absin, 1 mg/mL), for 60 min at 37 °C and then separated by gradient centrifugation. Spleen macrophages and lung macrophages were sorted after enzymatic digestion in Collagenase IV (Sigma) at 37 °C and RBC lysis for 1 min. Single cells of brain and intestine were sorted from mice after PBS wash and enzymatic digestion in tissue dissociation solution (Absin, 1 mg/mL), for 60 min at 37 °C and then separated by gradient centrifugation. Peripheral blood was added with 1 X red blood cell lysis buffer, and PBMCs were obtained after density gradient centrifugation. Cells were stained with Live/Dead fixable dead cell stain (Life technologies) prior to antibody staining.

### Isolation and culture of mouse bone marrow-derived macrophages

Mouse bone marrow-derived macrophages (BMDMs) were isolated from 6-8 weeks C57BL/6 mice. After the mice were euthanized, the femurs and tibias were rinsed with PBS using 27-gauge needles to obtain bone marrow cells. Then the collected cells were treated with red blood cell lysis buffer and centrifuged. Subsequently, the cells were cultured in DMEM medium supplemented with 10% FBS and M-CSF (50 ng/mL, Sino Biology). The culture medium was changed every 2-3 days, and mouse BMDMs were obtained after 7 days.

### scRNA-seq processing and filtering

The raw reads obtained from sequencing were aligned to the mouse genome (GRCm38/mm10). The unique molecular identifiers (UMIs) were estimated using Cell Ranger software (v3.1.0). The aligned features were subsequently loaded and processed using the Seurat package (v4.0.2) in R version 4.0.5. To filter out low-quality cells, cells were excluded if they expressed fewer than 200 genes or if more than 15% of their expressed genes were mitochondrial genes. Potential doublets were removed using DoubletFinder (v2.0.6) (PCs = 1:20, pN = 0.25, pK = 0.02).

### scRNA-seq normalization and clustering

After performing data preprocessing and quality control, the Seurat package was used for data normalization. The “NormalizeData” function was applied, utilizing “LogNormalize” as the normalization method with a scale factor of 100,000 (scale.factor=100000). To identify variable genes, the “FindVariableFeatures” function was utilized. Integration of the scRNA-seq data from different tissues was performed using the “FindIntegrationAnchors” function, which aligns and merges datasets from different sources. The scaled gene expression data were projected onto principal components (PCs), and the first 30 PCs capturing the most significant sources of variation were selected for further analysis. Non-linear dimensionality reduction was performed using Uniform Manifold Approximation and Projection (UMAP) to visualize and cluster cells based on gene expression similarities. Clustering was conducted using the “FindNeighbors” and “FindClusters” functions, which assign cells into distinct clusters based on their expression patterns. Marker genes specific to each cluster were identified using the “FindAllMarkers” function to find genes differentially expressed between clusters. To account for batch effects between scRNA-seq data form different tissues, batch correction was performed using Canonical Correlation Analysis (CCA). This method identifies linear combinations of features that are maximally correlated across different datasets, preserving the shared correlation structure. Finally, the data batches were pooled into a single object for downstream analyses, ensuring the shared structure is maintained across all samples.

### scRNA-seq differential gene expression analysis

To identify the signature genes of each cell type, the Seurat package was used, specifically the “FindAllMarkers” and “FindMarkers” functions. The “FindMarkers” function was utilized to identify signature genes by comparing the cell type of interest with another specific group of cells.

### ATAC-seq and CUT&Tag analysis

Bowtie2 was used to align the sequences to the mouse genome and generate bam files. After deprived of PCR duplicates using Picard tools, Deeptools (3.3.1) bamCoverage (CPM normalized and extended reads) was used to generate bigwig files from bam files. MACS2 (v2.2.5) was used for peak calling and to generate bed files from aligned reads. Our parameter settings are: P-value <0.05, shift is -100, extsize is 200 and nomodel, the remaining parameters are default. The HOMER (v4.10.0) annotatePeaks.pl was used to annotate the peaks. Binding peaks derived from bigWig files were visualized using IGV (2.6.2). For ATAC-seq, the heatmap profiles of the control group and *Id2* knockout peaks were drawn by plotHeatmap of Deeptools. The quantification plots of the different clusters of control and* Id2* knockout were summarized by plotProfile of Deeptools.

### Statistics

All experiments were carried out with at least three biological replicates. Data shown in column graphs represent the mean ± s.d., as indicated in the Figure legends. When normality could be assumed, Student's t-test, One-way ANOVA, or Two-way ANOVA analysis was used to compare difference between two groups as indicated in the Fig. legends. Statistical analysis was performed with GraphPad Prism10.

### Gene ontology (GO) analysis

For scRNA-seq, genes with P<0.05 and absolute value of |log2FC|≥1 were used for Gene ontology (GO) analysis, which was performed using the web tool: DAVID (http://david.adcc.ncifcrf.gov/). For ATAC-seq, we performed GO analysis on genes with differential binding peaks (FDR<0.05) identified by DiffBind. GO terms with P < 0.05 were determined to be statistically significant.

## Results

### A scRNA-seq atlas of multi-tissue macrophages under steady state and sepsis

**Figure 1:**
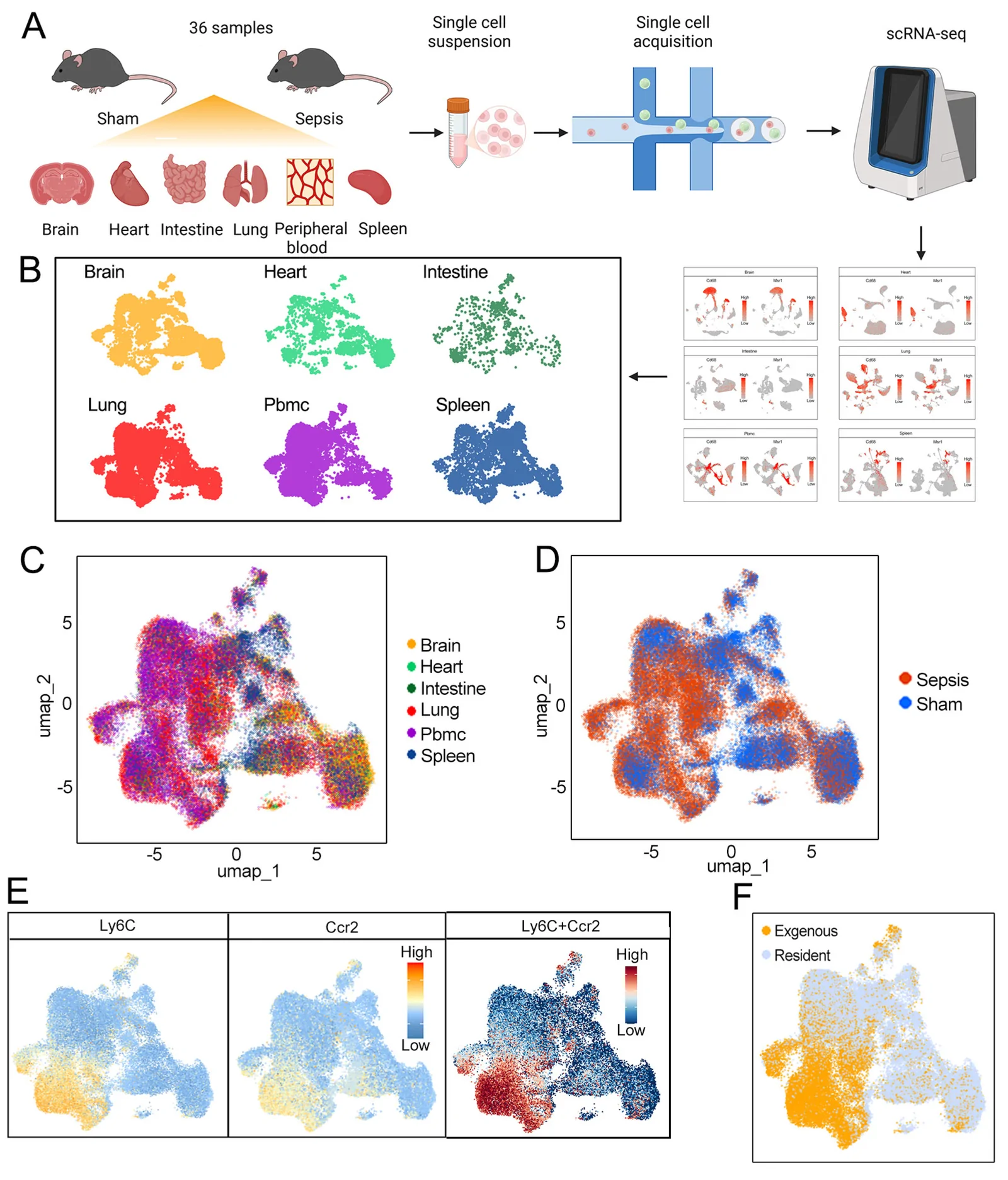
** Construction of single-cell atlas of sepsis and steady-state multi-tissue macrophages.** (A) The experimental workflow. (B) Macrophages in the brain, heart, intestine, lung, spleen, and peripheral blood mononuclear cells. (C and D) A scRNA-seq atlas of multi-tissue macrophages (C) under steady-state and sepsis (D). (E and F) According to the expression of *Ly6C* and *Ccr2* (E), macrophages are classified into exogenous and resident types (F).

In this study, we constructed sepsis model mice and used sham operated mice as controls to perform scRNA-seq on the brain, heart, small intestine, lung, peripheral blood, and spleen (Fig. 1A, Supplementary Table 1). After quality control, unsupervised clustering was performed on cells that meet the quality standards. Then we screened 52495 macrophages/monocytes from 36 samples of these 6 tissue libraries for downstream analysis based on the expression of *Cd68* and *Msr1*^15^, ^16^ (Fig. S1). For a comparative analysis, we merged PBMCs with macrophages from the other five tissues. Finally, we got a scRNA-seq atlas of multi-tissue macrophages under steady-state and sepsis. (Fig. 1B-D). According to the expression of *Ly6C* and *Ccr2*^17^, ^18^, macrophages of brain, heart, small intestine, lung and spleen can be further divided into tissue-resident and exogenous (Fig. 1E, F).

### Transcriptional heterogeneity of resident-macrophages between tissues under steady state

**Figure 2:**
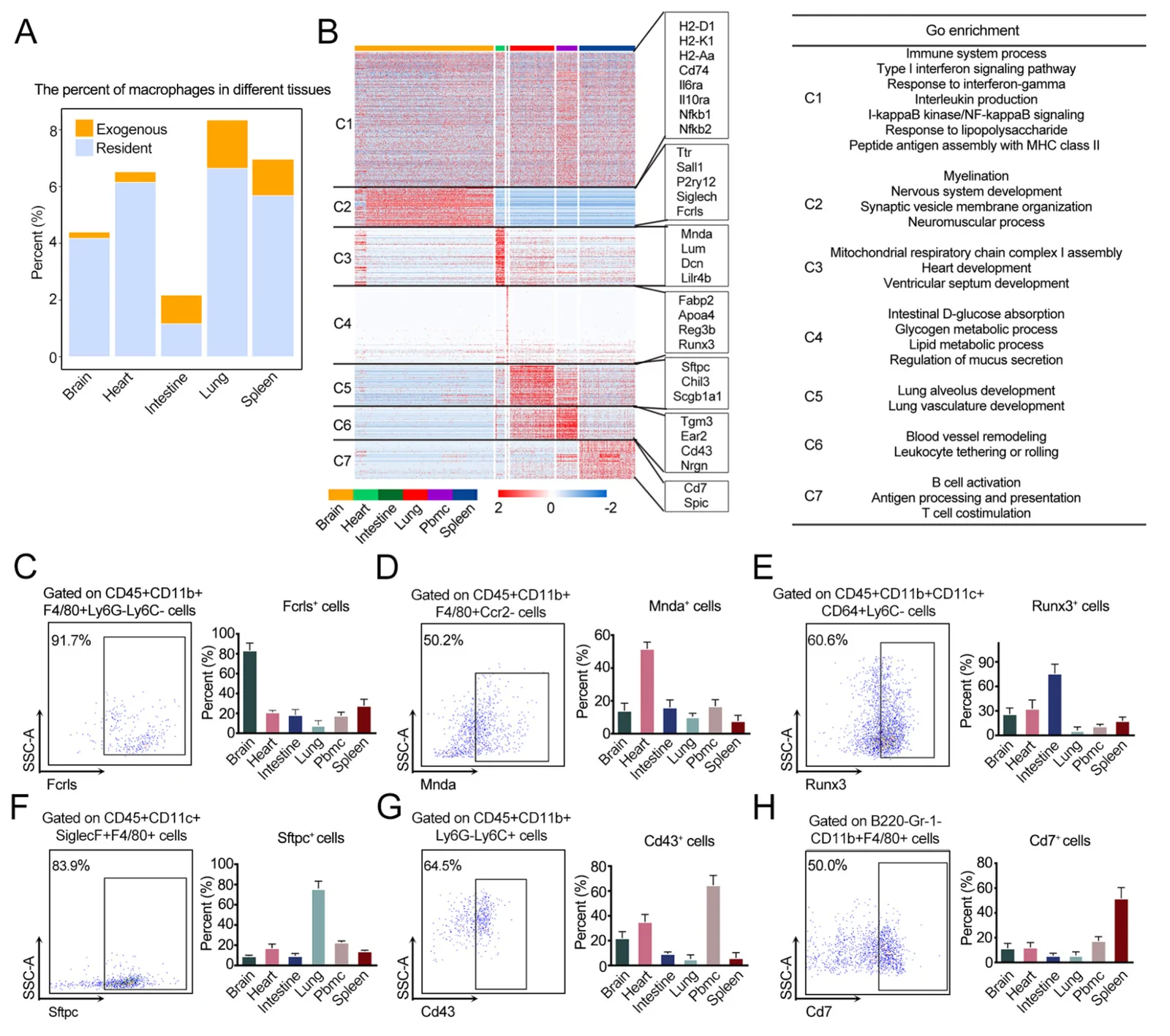
** Transcriptional heterogeneity of resident-macrophages between tissues in steady state.** (A) The percent of macrophages in different tissues under steady state. (B) The expression of consistent and specific genes between macrophages in peripheral blood and different tissues (left), and corresponding functional enrichment analysis (right). (C) The proportion of *Fcrls^+^* cells in macrophages of different tissues. (D) The proportion of *Mnda^+^* cells in macrophages of different tissues. (E) The proportion of *Runx3^+^* cells in macrophages of different tissue. (F) The proportion of *Sftpc^+^* cells in macrophages of different tissues. (G) The proportion of *Cd43^+^* cells in macrophages of different tissues. (H) The proportion of *Cd7^+^* cells in macrophages of different tissues.

Under steady-state, the proportion of macrophages in each tissue (only border-associated macrophages were counted for brain samples) is approximately 2%-8%. Compared with the brain and heart, exogenous monocytes account for a higher proportion in the intestine and lung (Fig. 2A). Under homeostasis, exogenous monocytes account for 40%-50% of intestinal macrophages. It's reported that macrophage pool in adult small intestine require constant replenishment by circulating monocytes to maintain normal numbers even in steady-state^19^, which consistent with our results. As the core organ of the respiratory system, the lung may need to recruit a large number of exogenous monocytes to defend against pathogens and foreign substances entering the respiratory tract.

To investigate the consistency and heterogeneity of resident-macrophages transcription among tissues (microglia are contained) and PBMCs, we divided genes into a conserved module (C1) and six tissue-specific modules (C2-C7). C1 genes showed high level of conserved expression among tissues, such as MHC II genes (*H2-Aa*, *H2-D1*,* H2-K1*, *Cd74*, etc.), *Il6ra*, *Il10ra*, *Nfkb1*, *Nfkb2* and so on. The consistent expression pattern of these genes suggests antigen presentation, interleukin production and LPS response pathways are functionally conserved across all tissue macrophages (Fig. 2B). Many genes are tissue-specific, including some transcription factors (TFs). C2 genes are specifically expressed in brain, such as *Ttr*, *P2ry12*, *Siglech*, *Fcrls* and TF *Sall1*. These genes play an important role in myelination, nervous system development and neuromuscular process (Fig. 2B, C; Fig. S2A, S3A). C3 genes showed higher expression in heart, such as *Mnda*, *Lum*, *Dcn* and *Lilr4b*, which involved in heart development (Fig. 2B, D; Fig. S2B, S3B). Genes *Fabp2*, *Apoa4*, *Reg3b* and TF *Runx3* in C4, had higher expression in intestine, related to intestinal absorption and metabolism (Fig. 2B, E; Fig. S2C, S3C). C5 genes were lung specific genes, such as* Sftpc*, *Chil3* and *Scgb1a1*, are important in lung development (Fig. 2B, F; Fig. S2D, 3D). C6 genes showed higher expression in PBMCs, including *Tgm3*, *Ear2*, *Cd43* and *Nrgn*, involved in blood vessel development (Fig. 2B, G; Fig. S2E, 3E). C7 genes *Cd7* and TF *Spic* highly expressed in spleen (Fig. 2B, H; Fig. S2F, 3F), are mainly involved antigen processing and presentation. In summary, resident macrophages isolated from different tissues possess unique gene expression signatures, which play an important role in maintaining tissue function and homeostasis.

### Diversity of functional resident-macrophage subtypes between tissues in steady state

**Figure 3:**
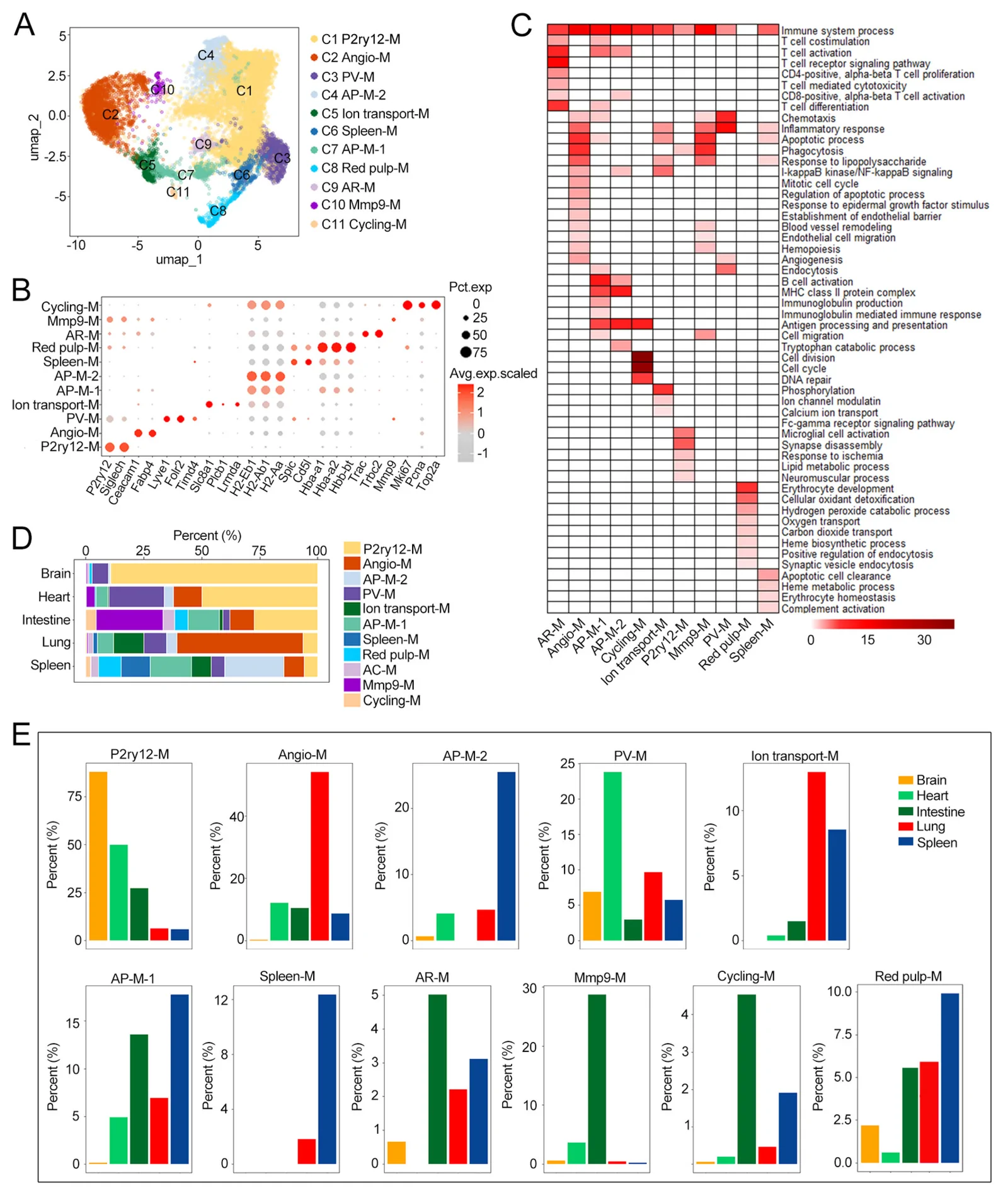
** Diversity of functional resident-macrophage subtypes between tissues in steady state.** (A) Identification of functional subtypes of macrophages under steady-state. (B) Marker genes of different functional subtypes. (C) The consistency and specificity of functions among different subtypes of macrophages. (D) The proportion of the different functional subtype in each tissue. (E) The proportion of the each functional subtype in different tissues.

To investigate the consistency and heterogeneity of functional subtypes of macrophages in different tissues under homeostasis, we applied unsupervised clustering to all steady-state resident macrophages and obtained 11 cell clusters. We further defined them as 10 functional subtypes according to their gene expression patterns (Fig. 3A, B). There are *P2ry12* high macrophages (P2ry12-M) (*P2ry12*, *Siglech*)^20^, ^21^, angiogenic macrophages (Angio-M) (*Ceacam1*, *Fabp4*)^22^, perivascular macrophages (PV-M) (*Lyve1*, *Folr2*, *Timd4*)^23^, ion transport macrophages (Ion transport-M) (*Slc8a1*, *Plcb1*, *Lrmda*)^24^, antigen presentation macrophages (AP-M) (*H2-Aa*, *H2-Ab1*, *H2-Eb1*), spleen specific macrophages (Spleen-M) (*Spic*, *Cd5l*), red pulp macrophages (Red pulp-M) (*Hba-a1*, *Hba-a2*, *Hbb-bt*), antigen recognition macrophages (AR-M) (*Trac*, *Trbc2*), *Mmp9* high macrophages (Mmp9-M) (*Mmp9*), and cycling macrophages (Cycling-M) (*Mki67*, *Pcna*, *Top2a*). The differential gene expression patterns determine the distinct functions of these subtypes (Fig. 3C).

Quantitative statistics revealed obvious tissue tropism differences among these resident macrophage subtypes (Fig. 3D, E). The proportion of PV-M in heart is significantly higher than in other tissues. It's reported that, PV-M can regulate smooth muscle cell collagen through hyaluronic acid to maintain vascular tension^25^. Our data indicated that as an organ with a dense vascular network, the heart has a large number of macrophages distributed around blood vessels to regulate vascular tension and homeostasis. Compared to the other tissues, the small intestine contains a higher proportion of Mmp9-M. Matrix metalloproteinases (Mmps) mainly involved in extracellular matrix degradation^26^, which may provide nutrients and space for the rapid proliferation of intestinal cells. Compared with AP-M1, AP-M2 mainly exist in the spleen, Red pulp macrophages highly express hemoglobin and have the function of clearing aging erythrocytes, were reported to be mainly distributed in the red pulp region of the spleen^27^. Our study found that red pulp-like macrophages also exist in trace amounts in the brain, heart, and small intestine, implying partial conservation of erythrocyte clearance function across organs.

Surprisingly, we detected a certain proportion of P2ry12-M in heart and intestine (Fig. 3D, E). *P2ry12* has been reported to be a marker of microglia and also been detected in the heart and intestine (Fig. S4A). Microglia were previously considered a macrophage subset exclusive to the central nervous system. Nevertheless, recent studies have identified cell populations carrying microglia-like transcriptional profiles in fetal skin, testis and heart tissue^20^. It's also reported that neuron-associated macrophages exist in intestine which highly express microglial signature genes such as *P2ry12*^28^.

### Sepsis induces consistent transformation of macrophages from different tissues to glycolytic type

**Figure 4:**
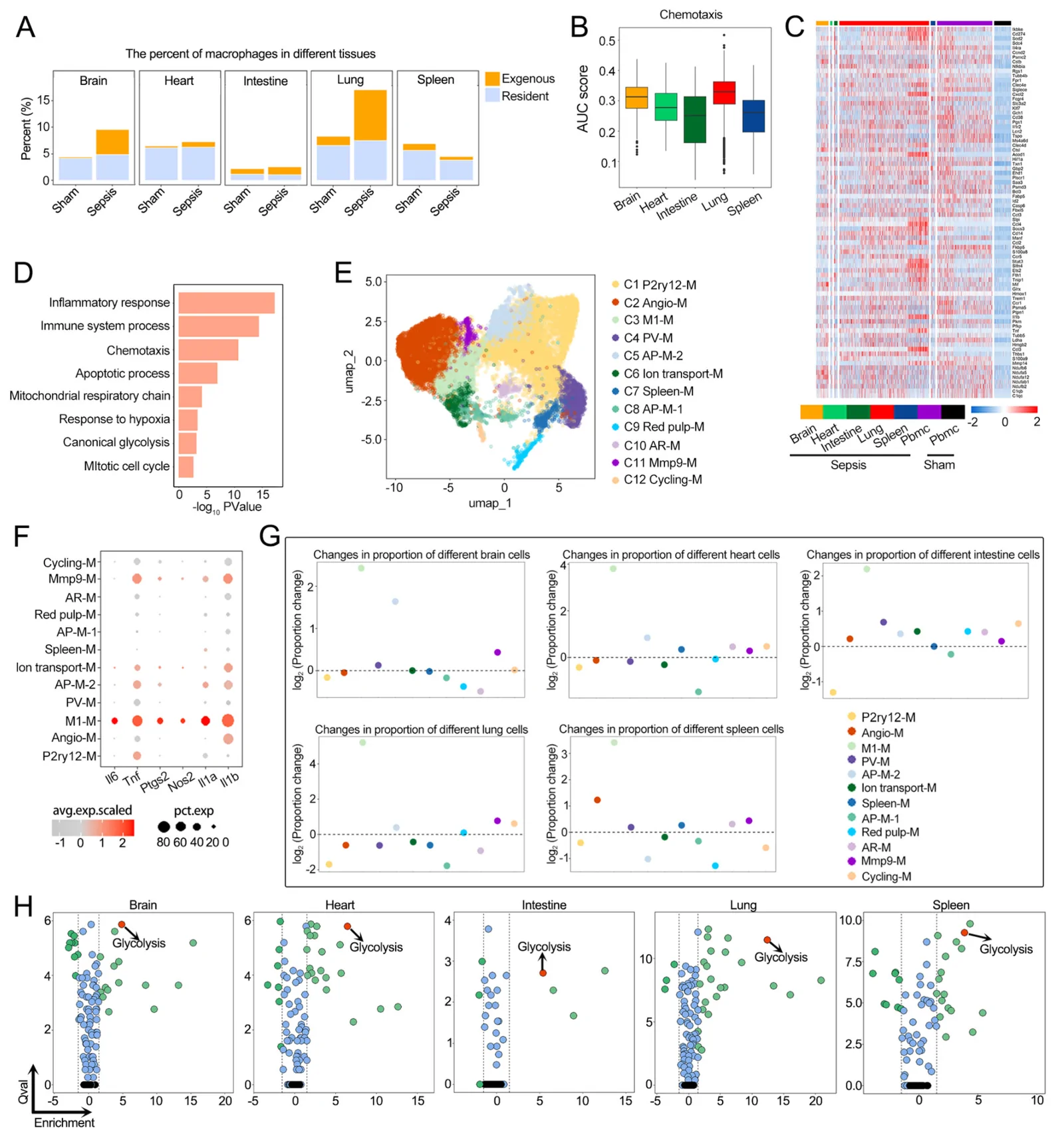
** Changes in exogenous and resident macrophages after sepsis.** (A) Changes in the proportion of exogenous and resident macrophages in different tissues after sepsis. (B) AUC scores of chemokine related genes in exogenous macrophages from different tissues. (C) Compared with PBMCs in steady state, genes consistently upregulated in exogenous macrophages of all tissues after sepsis. (D) Functional enrichment analysis of upregulated genes in (C). (E) Combining resident macrophages of steady-state and sepsis, a cell cluster of M1 macrophages was identified after sepsis compared to steady-state in (Figure 3A). (F) Marker genes in M1 macrophage. (G) Changes in the proportion of different functional subtypes of macrophages after sepsis. (H) Single cell pathway analysis (SCPA) of metabolic pathway changes in different tissues after sepsis.

Previous studies have shown that exogenous monocytes will migrate to the lesion under inflammatory stimuli such as infection and injury^29^, ^30^. We calculated the proportions of exogenous monocytes after sepsis. Results show that all tissues recruit a large number of exogenous monocytes after sepsis, and the lungs being the most significant (Fig. 4A). Gene signature scoring based on chemotaxis-related transcripts demonstrated that exogenous monocytes in the lungs do exhibit stronger chemotaxis (Fig. 4B). This may indicate that inflammatory response caused by sepsis is most severe in the lung. A systematic comparison of exogenous monocytes and PBMCs revealed that 80 genes were uniformly upregulated in all tissues after sepsis (Fig. S4B, C). Many of them involved in inflammatory response (*Lcn2*, *Saa3*, *Tnf*, *Stat3*, et al.) and glycolysis (*Pkm*, *Pfkp* and *Ldha*) (Fig. 4C, D; Fig. S4D). This indicates a consistent shift towards glycolysis in the metabolic patterns of exogenous monocytes in all tissues after sepsis.

Correlation analysis of transcriptome profiles indicated that gene expression patterns of resident macrophages subtypes are more similar after sepsis (Fig. S5A, B), indicating their phenotypes tend to be more consistent. To capture sepsis-induced functional and phenotypic shifts, we integrated resident macrophages from both steady-state and septic groups for unsupervised clustering. A new cluster of M1 type macrophage (M1-M) with high expression of inflammatory factors such as* Il6* and *Nos2* were identified after sepsis (Fig. 4E, F). The proportion of Mmp9-M increases while AP-M-1 decreases in most tissues after sepsis (Fig. 4G). In addition, we conducted differential gene expression analysis for each cell subtype to screen sepsis-altered transcripts. Results showed that pro-inflammatory genes such as *Saa3*, *Lcn2*, and *Lrg1*, as well as extracellular matrix degradation gene *Mmp14*, are upregulated in many subtypes, while anti-inflammatory gene *Hpgd* and antigen presentation genes *H2-Eb1*, *Fcer2a* are downregulated after sepsis (Fig. S5C). The function of macrophages is related to cellular metabolism. Metabolic pathway enrichment analysis revealed universal upregulation of glycolysis in all tissue-resident macrophages after sepsis (Fig. 4H). Collectively, these data demonstrate that sepsis drives resident macrophages to shift toward a glycolytic type with enhanced pro-inflammatory and extracellular matrix degradation capacity and impaired antigen presentation function.

### Comparative analysis of differentially expressed genes revealed that Pkm2 is consistently upregulated in multi-tissue glycolytic macrophages after sepsis

To explore the functional changes of glycolytic macrophages, we performed pairwise differential expression analysis between steady-state and septic macrophages for each tissue and carried out cross-tissue comparative screening (Fig. S6A). We first analyzed the down-regulated genes and found that the genes related to homeostasis maintenance were specifically down-regulated in tissues. Such as *Hexa*, *Sall1* and *Fcrls,* which are considered to be crucial for maintaining the function of microglia^9^, ^31^ (Fig. S6B, E). There were 8 genes downregulated in all five tissues, including 7 antigen presenting genes (*Cd74*, *H2-Aa*, *H2-Ab1*, *H2-DMa*, *H2-DMb1*,* H2-Eb1* and *Ighm*) and *Selenop* which associated with M2 macrophage polarization^32^ (Fig. S6C-E). This consistent transcriptional repression indicates that impaired antigen presentation and weakened anti-inflammatory capacity are universal features of macrophages in all tissues.

**Figure 5:**
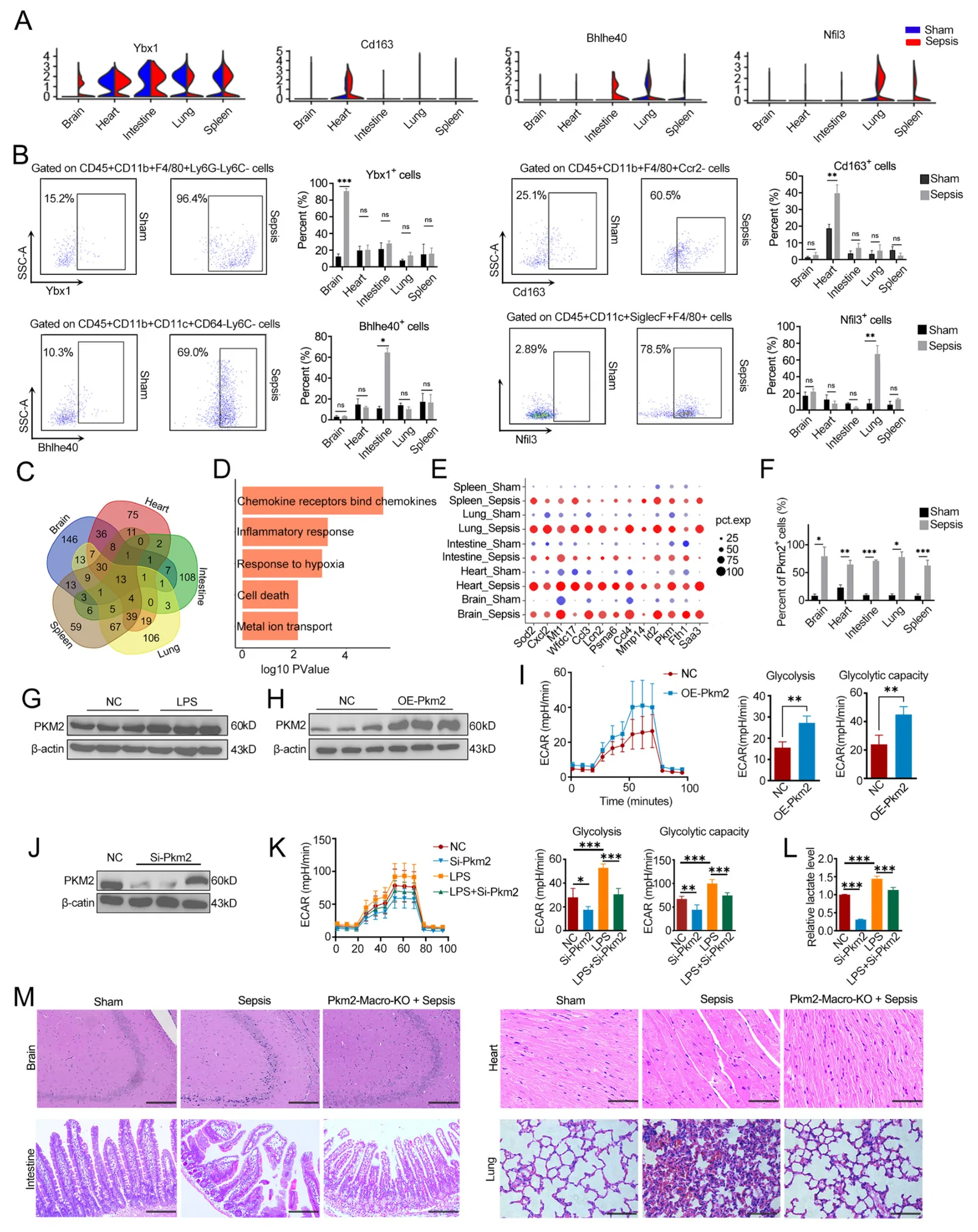
** Comparative analysis of differentially expressed genes revealed that Pkm2 is consistently upregulated in multi-tissue macrophages after sepsis.** (A) Violin plot shows the expression of *Ybx1*, *Cd163*, *Bhlhe40* and *Nfil3* in different tissues of sham and sepsis. (B) Flow cytometry revealed the proportions of *Ybx1^+^*, *Cd163^+^*, *Bhlhe40^+^* and *Nfil3^+^* cells in resident macrophages of different tissues from mice with sham and sepsis. (C) Venn plot shows the up-regulated in different tissues. (D) Functional enrichment analysis of 13 overalp genes in (C). (E) 13 genes consistently up-regulated in different tissues after sepsis. (F) The percent of *Pkm2*^+^ macrophages in different tissues of sham and sepsis. The data were analyzed by Student's *t* test. *P < 0.05, **P < 0.01 and ***P < 0.001. (G) Western blot of PKM2 protein in normal control and 1 µg/mL LPS stimulated RAW264.7 cells (n=3). (H) Western blot of PKM2 protein in normal control and RAW264.7 cells with Pkm2-expressing adenovirus (n=3). (I) Extracellular acidification rate (ECAR). All data are expressed as mean ± SEM (n = 3). The data were analyzed by Student's *t* test. *P < 0.05, **P < 0.01 and ***P < 0.001. (J) Western blot of PKM2 protein in normal control and RAW264.7 cells using siRNA against PKM2 (n=3). (K) ECAR of cells, all data are expressed as mean ± SEM (n = 3). The data were analyzed by Student's *t* test. *P < 0.05, **P < 0.01 and ***P < 0.001. (L) Lactate levels compared to the normal control group, all data are expressed as mean ± SEM (n = 3). The data were analyzed by Student's *t* test. *P < 0.05, **P < 0.01 and ***P < 0.001. (M) H&E images of brain, heart, intestine and lung from sham, septic and Pkm2-Macro-KO septic mice, scale bars, 100 µm, n=5.

Next, we characterized sepsis-induced upregulated genes and found that some genes show tissue-specific upregulation. Compared with other tissues, the expression of *Ybx1*, which promotes glycolysis^33^, was significantly increased in the brain (Fig. 5A, B; Fig. S7A). *Cd163* is significantly upregulated in the heart (Fig. 5A, B; Fig. S7B), and it is known that *Cd163^+^* macrophages have the function of promoting angiogenesis^34^. *Bhlhe40* was significantly upregulated in intestine (Fig. 5A, B; Fig. S7C), which also has a promoting effect on glycolysis^35^. The pro-inflammatory factor *Nfil3* is significantly upregulated in the lung (Fig. 5A, B; Fig. S7D). The above results reflected different ways in which tissues respond to sepsis by resident macrophages.

To screen potential targets for the treatment of multi-tissue damage, we focused on genes with consistent upregulation across all five examined tissues. There were 13 genes upregulated in all five tissues after sepsis (Fig. 5C), GO analysis showed that these genes mainly related to chemokine binding and inflammatory responses (Fig. 5D). In addition, the glycolytic gene *Pkm*, as well as the transcription regulatory factor *Id2*, were also significantly upregulated (Fig. 5E).

It's known that *Pkm* generates two variants through alternative splicing, *Pkm1* and *Pkm2*^36^. Among them, *Pkm2* has been reported to play an important role in the Warburg effect in cancer^37^. In septic mice, the proportion of Pkm2^+^ macrophages significantly increased in all tissues (Fig. 5F; Fig. S8A). We analyzed the role of *Pkm2* in sepsis at both the cellular and mouse levels. In RAW264.7 cells, the expression of *Pkm2* was up-regulated after lipopolysaccharide (LPS) treatment (Fig. 5G). We constructed *Pkm2*-overexpressing macrophages by adenovirus (Fig. 5H), and found the level of glycolysis was significantly increased (Fig. 5I). While inhibition of *Pkm2* by siRNA can limit the formation of LPS induced glycolytic macrophages and lactate production (Fig. 5J-L). In septic mouse, to investigate the role of glycolytic macrophages induced by *Pkm2* in multi-tissue damage, we generated resident macrophage-specific *Pkm2* conditional knockout (Pkm2-Macro-KO) mice by crossing* Cx3cr1*CreER and *Pkm2*^fl/fl^ strains. Results showed that the tissue damage of hippocampus, myocardium, intestinal epithelium and alveoli caused by sepsis was significantly alleviated in the Pkm2-Macro-KO mice (Fig. 5M).

### *Id2* induces macrophage metabolic reprogramming through modulating the chromatin accessibility of *Pkm2*

**Figure 6:**
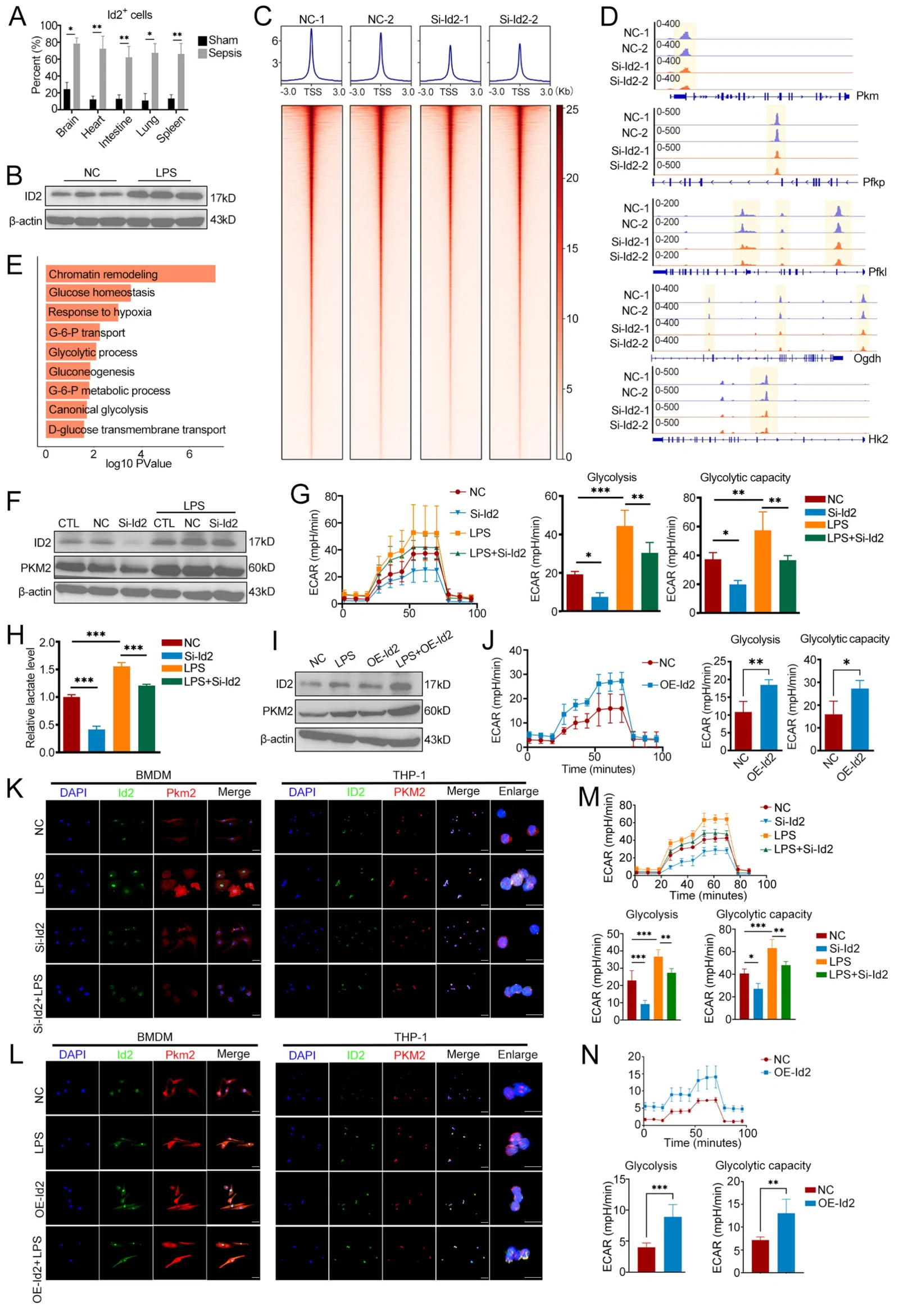
***Id2* induces macrophage metabolic reprogramming through modulating the chromatin accessibility of *Pkm2*.** (A) The percent of *Id2^+^* macrophages in different tissues of sham and sepsis. (B) Western blot of ID2 protein in normal control and LPS stimulated RAW264.7 cells (n=3). (C) ATAC-seq profiles of normal control and RAW264.7 cells transfected with si-Id2. (D) Integrative Genomics Viewer tracks display ATAC-seq reads along the indicated genes. (E) Functional enrichment analysis of genes with reduced chromatin accessibility. (F) Western blot of ID2 and PKM2 in RAW264.7 cells transfected with si-Id2. (G) ECAR of cells, all data are expressed as mean ± SEM (n = 3). The data were analyzed by Student's *t* test. *P < 0.05, **P < 0.01 and ***P < 0.001. (H) Lactate levels compared to the normal control group, all data are expressed as mean ± SEM (n = 3). The data were analyzed by Student's *t* test. *P < 0.05, **P < 0.01 and ***P < 0.001. (I) Western blot of ID2 and PKM2 in RAW264.7 cells with Id2-expressing adenovirus or stimulated with LPS. (J) ECAR of cells with Id2-expressing adenovirus (n=3). The data were analyzed by Student's *t* test. *P < 0.05, **P < 0.01 and ***P < 0.001. (K) Representative images illustrating the expression level of *Pkm2* (red) after knocking down *Id2* (green) with siRNA in mouse BMDMs (left) and THP-1 cells (right) with DAPI-stained nuclei (blue). Each dot represents one experiment, scale bars, 10µm, n =3. (L) Representative images illustrating the expression level of *Pkm2* (red) after overexpressing *Id2* (green) via adenovirus in mouse BMDMs and THP-1 cells with DAPI-stained nuclei (blue). Each dot represents one experiment, scale bars, 10µm, n =3. (M) ECAR of mouse BMDMs, all data are expressed as mean ± SEM (n=4). The data were analyzed by Student's *t* test. *P < 0.05, **P < 0.01 and ***P < 0.001. (N) ECAR of mouse BMDMs with Id2 overexpression via adenovirus, all data are expressed as mean ± SEM (n=5). The data were analyzed by Student's *t* test. *P < 0.05, **P < 0.01 and ***P < 0.001.

Previous studies have shown that the expression of *Pkm2* is often regulated by some transcription factors^38^. Studies have shown that hypoxia can rapidly induce the transcription of *Id2*. As a transcription regulatory factor, *Id2* has been shown to regulate genes expression by altering chromatin accessibility^39^, ^40^. As mentioned earlier, *Id2* and *Pkm2* are consistently upregulated in all tissues (Fig. 5E). In addition, we further re-analyzed single-nucleus RNA-seq data of hippocampus from sepsis patients and age-matched controls in GEO database^41^ (GSE307512). The results showed that *ID2* was upregulated in microglia and monocytes from the hippocampus of sepsis patients, which consistent with the observations in mice (Fig.S8B-D). Flow cytometry revealed that the proportion of* Id2^+^* resident macrophages significantly increased in all tissues after sepsis (Fig. 6A and Fig. S8E). In cellular level, the expression of *Id2* was upregulated in macrophages treated with LPS (Fig. 6B). To investigate whether there is a regulatory relationship between *Id2* and *Pkm2*, and to explore the role of *Id2* in macrophages, we performed ATAC-seq. Overall, *Id2* knockout led to reduced chromatin accessibility in macrophages (Fig. 6C). We identified 123 up-regulated and 3300 down-regulated peaks (Fig. S9A, Supplementary Table 2) and found the accessibility of many glycolytic genes such as *Pkm*, *Pfkp*, *Pfkl*, *Ogdh*, and *Hk2* was reduced (Fig. 6D). Functional enrichment analysis showed that genes with reduced chromatin accessibility mainly involved in glucose metabolism, including glycolysis and gluconeogenesis (Fig. 6E). To further verify the regulatory effect of *Id2* on *Pkm2*, we knocked down the expression of* Id2* in RAW264.7 cells using siRNA to observe the expression of *Pkm2*. The results indicated that inhibition of *Id2* downregulated the expression of *Pkm2* (Fig. 6F), limiting the formation of glycolytic macrophages and lactate production(Fig. 6G, H). While, overexpression of *Id2* upregulated *Pkm2* and enhanced macrophage glycolysis (Fig. 6I, J).

Furthermore, we extended our investigation to mouse BMDMs and THP-1 cells. We then knocked down *Id2* in these two types of cells using siRNA, and the results were consistent with those observed in RAW264.7. The knockdown of *Id2* downregulated *Pkm2* and effectively inhibited the LPS-induced upregulation of *Pkm2* in both BMDMs and THP-1 cells (Fig. 6K). In contrast, when BMDMs and THP-1 cells were infected with adenovirus to overexpress *Id2*, the expression of *Pkm2* increased (Fig. 6L). Subsequently, we detected the glycolysis levels of BMDMs, and found that *Id2* downregulation significantly inhibited the glycolysis of macrophages under both steady-state and LPS treatment (Fig. 6M). While overexpression of *Id2* could enhance the glycolysis levels of BMDMs (Fig. 6N). Therefore, we conclude that *Id2* can effectively regulate macrophage glycolysis through *Pkm2* during sepsis.

It's reported that *Id2* lacks a basic DNA-binding domain, but can form a heterodimer with bHLH transcription factors such as E2-2 through its HLH dimerization domain to regulate genes expression^42^-^44^. In this study, we found that *Id2* and transcription factor 4 (*Tcf4*, also known as E2-2) were co-localized in macrophages, which consistent with previous reports (Fig. S9B). To investigate whether *Tcf4* is the partner of *Id2* in regulating *Pkm2*, we conducted CUT&Tag assay (Supplementary Table 3). The results showed that *Tcf4* had significant binding peaks on glycolytic genes such as *Pkm*, and the binding regions overlapped highly with the regulatory regions of *Id2* (Fig. S9C). Additionally, through Co-IP experiments, we observed that Tcf4 could bind to Pkm2 (Fig. S9D). What's more, the expression of *Pkm2* decreased following the knockdown of *Tcf4* (Fig. S9E). Therefore, we can reasonably infer that the upregulation of *Id2* promoted the chromatin opening of glycolytic genes, and Tcf4 cooperates with *Id2* to regulate *Pkm2* transcription.

### The *Id2* inhibitor helichrysetin can significantly alleviate the multi-tissue damage caused by sepsis

**Figure 7:**
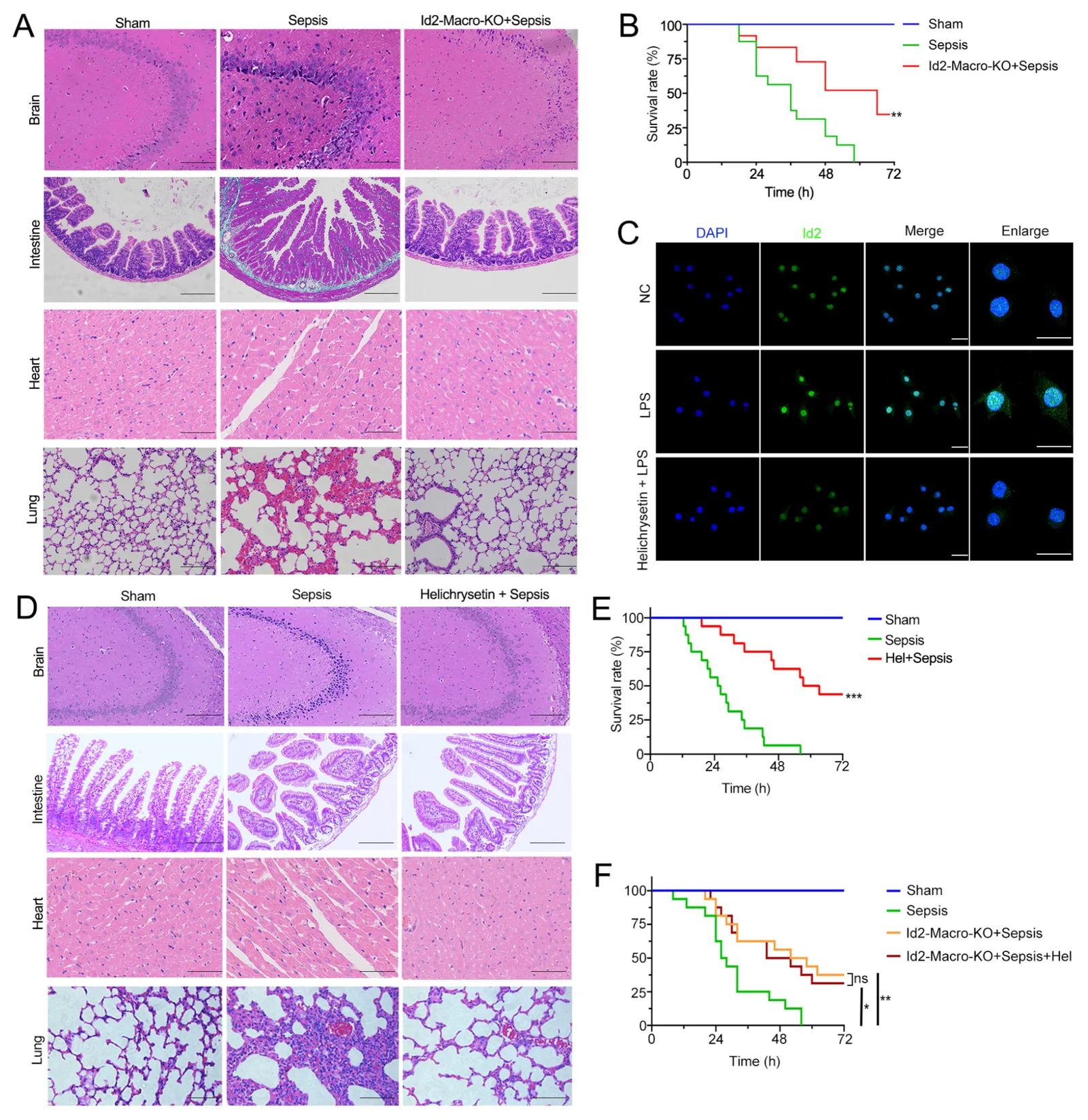
** The *Id2* inhibitor helichrysetin can significantly alleviate the multi-tissue damage caused by sepsis.** (A) H&E images of brain, heart, intestine and lung from sham, septic and Id2-Macro-KO septic mice, scale bars, 100µm, n=5. (B) 72-h survival rate and survival time of mice with sham, sepsis and Id2-Macro-KO+Sepsis (n=16 mice/group). The data were analyzed by the log-rank (Mantel‒Cox) test. *P < 0.05, **P < 0.01 and ***P < 0.001. (C) Representative images illustrating the expression level of *Id2* (green) in mouse BMDMs with DAPI-stained nuclei (blue) under three conditions: normal control, 1 µg/mL LPS-treated, and LPS-treated followed by 10 µM helichrysetin addition. Each dot represents one experiment, scale bars, 10µm, n =3. (D) H&E images of brain, heart, intestine and lung from sham, septic and helichrysetin treatment septic mice, scale bars, 100µm, n=5. (E) 72-h survival rate and survival time of sham, septic and helichrysetin treatment septic mice (n=16 mice/group). The data were analyzed by the log-rank (Mantel‒Cox) test. *P < 0.05, **P < 0.01 and ***P < 0.001. (F) 72-h survival rate and survival time of mice with sham, sepsis, Id2-Macro-KO+Sepsis, and Id2-Macro-KO+Sepsis+helichrysetin treatment (n=16 mice/group). The data were analyzed by the log-rank (Mantel‒Cox) test. *P < 0.05, **P < 0.01 and ***P < 0.001.

To further explore the role of *Id2*-mediated macrophage metabolic reprogramming in multi-tissue damage in sepsis, we generated resident macrophage-specific *Id2* conditional knockout (Id2-Macro-KO) mice by crossing *Cx3cr1*CreER mice with *Id2*^fl/fl^ mice. We established sepsis models with Id2-Macro-KO mice and found that, the damage of hippocampus, myocardium, intestinal epithelium and alveoli were significantly alleviated, and the survival rate was significantly increased (Fig. 7A, B). The results suggest that *Id2* can serve as a therapeutic target to effectively alleviate multiple tissue damage in sepsis and improve survival rates.

Helichrysetin is a natural product isolated from the flowers of helichrysum odoratissimum, and it has been reported to be an inhibitor of *Id2*^45^, ^46^. We observed that helichrysetin can significantly inhibited the LPS-induced upregulation of *Id2* in mouse BMDMs (Fig. 7C). To investigate whether helichrysetin can serve as a potential drug for the treatment of sepsis, we intraperitoneally injected helichrysetin into septic mice (10mg/kg). Results showed that in the septic mice treated with helichrysetin, the damage of hippocampus, myocardium, intestinal epithelium and alveoli was significantly alleviated (Fig. 7D). And helichrysetin can significantly improve the survival rate of septic mice (Fig. 7E). However, treatment with helichrysetin did not further increase the survival rate of Id2-Macro-KO septic mice (Fig. 7F),which indicated that the high expression of *Id2* was a key prerequisite for helichrysetin to exert its therapeutic effect on sepsis. The above results suggest that *Id2* can serve as a molecular target for the treatment of multi-tissue damage in sepsis, and helichrysetin can be a promising drug for the treatment of sepsis.

## Discussion

Under steady-state conditions, we classified tissue-resident macrophages into 10 functional subtypes based on their distinct gene expression profiles., These subtypes showed clear tissue-specific distribution, which reflecting adaptations to each tissue's unique physiological needs. For example, PV-M were more abundant in the heart, as a tissue with a dense distribution of blood vessels, the enrichment of PV-M in the heart is consistent with their known role in maintaining vascular homeostasis^47^, ^48^. We also found Mmp9-M were enriched in the small intestine. In homeostasis, *Mmp9* has been reported to play important role in intestinal goblet cell differentiation by moderately activating the Notch signaling pathway^49^. While in the intestinal inflammation model, the up-regulated *Mmp9* will degrade the extracellular matrix and disrupt the integrity of the basement membrane, which promoting crypt damage^50^. Based on the existing reports, we speculate that under the steady-state, Mmp9-M degrades the extracellular matrix, influencing signal transduction or providing nutrients and space for intestinal cell proliferation and differentiation. However, after sepsis, it will disrupt cell junctions and damage the basement membrane, which leads to intestinal barrier dysfunction. Collectively, these tissue-specific distribution and functional characteristics of resident macrophage subtypes demonstrate that tissue-resident macrophages serve as core guardians of host tissue homeostasis, and the subtype-specific tissue tropism is precisely shaped by the intrinsic structural and functional properties of distinct organs. After sepsis, our cross-tissue analysis further revealed the specific changes in different tissues. The lung showed the strongest recruitment of exogenous monocytes, which matches the existing perspective that lung is highly vulnerable to sepsis-induced acute respiratory distress syndrome (ARDS) due to robust immune cell infiltration^51^. Cardiac macrophages upregulated *Cd163*, a marker linked to angiogenic and tissue-repair functions in macrophages^34^, suggesting the heart prioritizes angiectasis during sepsis. Intestinal macrophages showed increased *Bhlhe40* expression, *Bhlhe40* was found to promote glycolysis in macrophages^35^, which potentially exacerbating intestinal barrier disruption. These differences highlight that sepsis elicits some tissue-specific responses, not a uniform systemic reaction, which is critical for understanding tissue-specific sepsis pathologies.

In this study, we found that sepsis induces a conserved glycolytic shift in macrophages across all tested tissues. The upregulation of glycolytic genes (*Pkm*, *Pfkp*, *Ldha*) and pro-inflammatory genes (*Lcn2*, *Saa3*, *Tnf*), consistent with the description of Warburg-like metabolism in pro-inflammatory macrophages^52^. Our key finding is the identification of the *Id2-Pkm2* axis as a central regulator of macrophage metabolic reprogramming in all tissues of sepsis. As a transcriptional regulatory factor, in recent studies, *Id2* has been revealed to regulate the accessibility of chromatin. For example, *Id2* increased chromatin openness of *Slamf6* and controlled Cd8^+^ T cell exhaustion^40^. Another study found that knockout of *Id2* in NK cells can promote chromatin openness of T cell related genes^39^. Our research found that *Id2* in macrophages can regulate the chromatin accessibility of glycolytic genes during sepsis. *Id2* has been reported to be regulated by *Hif1a*, there were two functional *Hif1a* binding sites in the *Id2* gene regulatory region, and *Hif1a* specifically binds to these sites to initiate *Id2* transcription^53^. Therefore, we hypothesize that hypoxic signals induce the upregulation of *Id2* after sepsis, promoting the chromatin opening of glycolytic genes. As a transcription factor of bHLH family, *Tcf4* can bind to *Id2*, acting as a mediator and combine with the chromatin opening area to regulate the transcription of glycolytic genes such as *Pkm2*. This newly identified link between *Id2* and *Pkm2* integrated transcriptional epigenetic control with metabolic reprogramming, substantially enhancing our mechanistic understanding of how macrophages switch to glycolysis during sepsis.

Although our current study does not focus on mitochondrial homeostasis regulation, it is a non-negligible and critical pathological issue that deserves close attention in the progression of sepsis. Accumulating studies have demonstrated that mitochondrial structural and functional disorders act as crucial inducers of macrophage phenotypic transformation during septic inflammation and are closely correlated with macrophage metabolic reprogramming. Abnormal activation of mitochondrial fission and suppression of mitophagy aggravate mitochondrial damage and cellular dysfunction under inflammatory stress, which profoundly affects the tissue adaptability of resident macrophages^54^. Targeted regulation of mitochondrial metabolic homeostasis, including modulation of intracellular malate and fumarate pools, can effectively restore mitochondrial fitness, activate protective mitophagy, and inhibit macrophage programmed necrosis, thereby limiting excessive inflammatory amplification^55^. In addition, natural bioactive components and nano-drug delivery systems exert potent protective effects against stress-induced myocardial and mitochondrial injury by regulating endoplasmic reticulum stress and mitochondrial quality surveillance, providing solid theoretical support for mitochondrial-targeted intervention strategies in sepsis^56^, ^57^.

Finally, we evaluated the therapeutic potential of targeting *Id2* using the helichrysetin. Our results showed intraperitoneal injection of helichrysetin improved septic mice survival and reduced multi-tissue damage. However, like many natural products, helichrysetin faces clinical translation challenges. Currently, as the inhibitor of *Id2*, helichrysetin is only used for scientific research. Its pharmacokinetics, pharmacodynamic biomarkers, and off-target effects have not been reported yet, and more exploration is still needed. Nevertheless, accumulating studies have indicated that natural antioxidants exhibit unique application advantages and great research potential in the treatment of various diseases by regulating redox imbalance^58^. In homeostasis, *Id2* plays a crucial role in the development of NK cells and the differentiation of Th17 cells^59^. Intestinal barrier damage is observed in Id2-null mice^60^. Therefore, when inhibiting *Id2* for a long time or completely, we must to consider whether it will lead to immunodeficiencies, and chronic toxicological studies are required in the future.

There are still some limitations in the current study. The CLP method used in this study is currently regarded as the "gold standard" for establishing sepsis models, with high reproducibility and stability. Although its pathological changes are highly similar to those in humans, due to the fact that human sepsis often accompanies underlying diseases, the mouse model is difficult to fully simulate the progression of human sepsis. Moreover, there are species differences in macrophage gene expression patterns and drug metabolism systems between mice and humans. These factors may lead to discrepancies between the results of mouse models and the clinical application in humans. Although some key findings of this study have been verified in THP-1 cells, future experiments still need to be conducted on tissue samples of human septic patients to enhance the clinical translatability of the current results.

Collectively, this study identifies the pan-tissue *Id2*-*Pkm2* axis governing macrophage glycolytic reprogramming across multiple organs and verifies the anti-septic organ-protective potential of helichrysetin, providing a novel therapeutic target and promising natural small-molecule candidate for translational intervention against sepsis-induced multiple organ injury. However, several critical pieces of supporting evidence limiting the clinical translational feasibility of our research scheme are still absent: comprehensive pharmacokinetic parameters, tissue distribution profiles and long-term systemic toxicological characteristics of helichrysetin have not been systematically characterized. In addition, the classic CLP mouse sepsis model carries inherent defects and cannot fully recapitulate the complex disease progression and diverse underlying comorbidities observed in human clinical sepsis. All these unresolved gaps merit thorough investigation in subsequent research.

## Supplementary Material

Supplementary figures and tables.

## Data Availability

The ATAC-seq and CUT&Tag data generated in this study are publicly available through the Gene Expression Omnibus (GEO) with the accession code GSE312985 and GSE312986. The scRNA-seq matrix data of macrophages are publicly available through the OMIX (https://ngdc.cncb.ac.cn/omix: accession No. OMIX013516).
