# Supplementary material for: Cross-tissue single-cell atlas reveals Id2 promotes sepsis-induced tissue damage by mediating macrophage metabolic reprogramming via Pkm2
Source: Int J Biol Sci. 2026 Aug 24;22(13):7471–86. doi: 10.7150/ijbs.136855 (PMC13540997; doi:10.7150/ijbs.136855)
Supplement: Supplementary file 1 [file ijbsv22p7471s1.zip › Supplementary materials/Supplementary figures.pdf]

Supplemental Materials

Cross-tissues single-cell atlas reveals Id2 promotes sepsis-induced tissue damage by mediating macrophage metabolic reprogramming via Pkm2

Supplementary figures: S1-S8

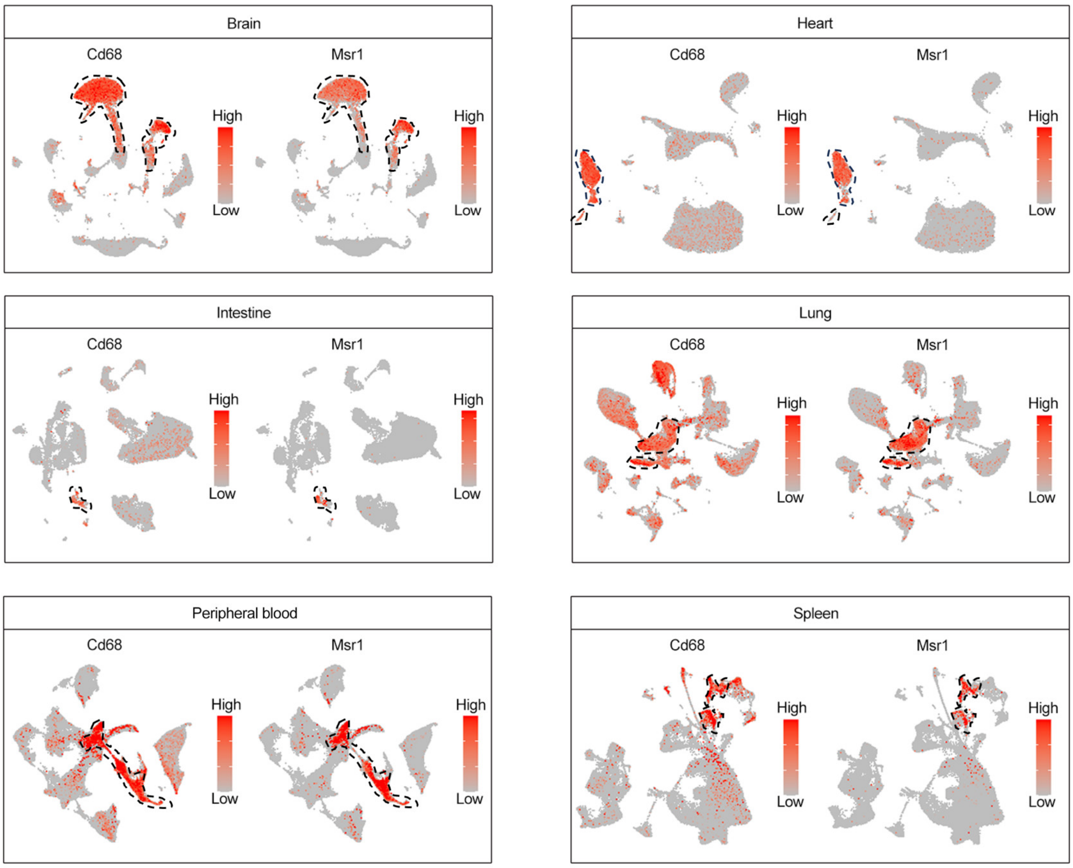

Figure S1. The expression of *Cd68* and *Msr1* in different tissues. Isolate macrophages from each tissue single-cell library based on the expression of *Cd68* and *Msr1*.

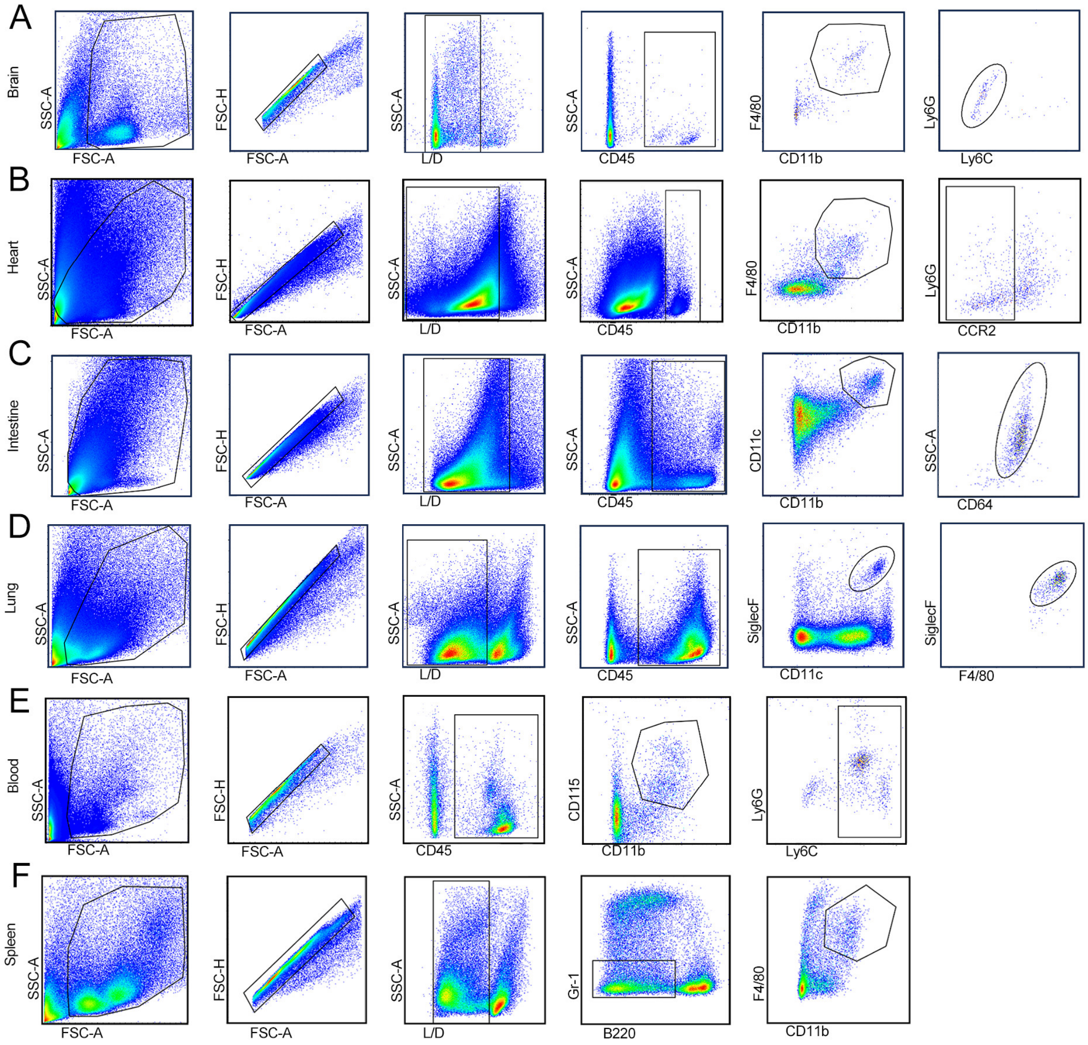

Figure S2. Defining and isolating tissue-resident macrophages and PBMCs. Flow cytometry sorting strategy for resident macrophages of brain (A), heart (B), intestine (C), lung (D), and spleen (F). (E) Flow cytometry sorting strategy for peripheral blood mononuclear cells.

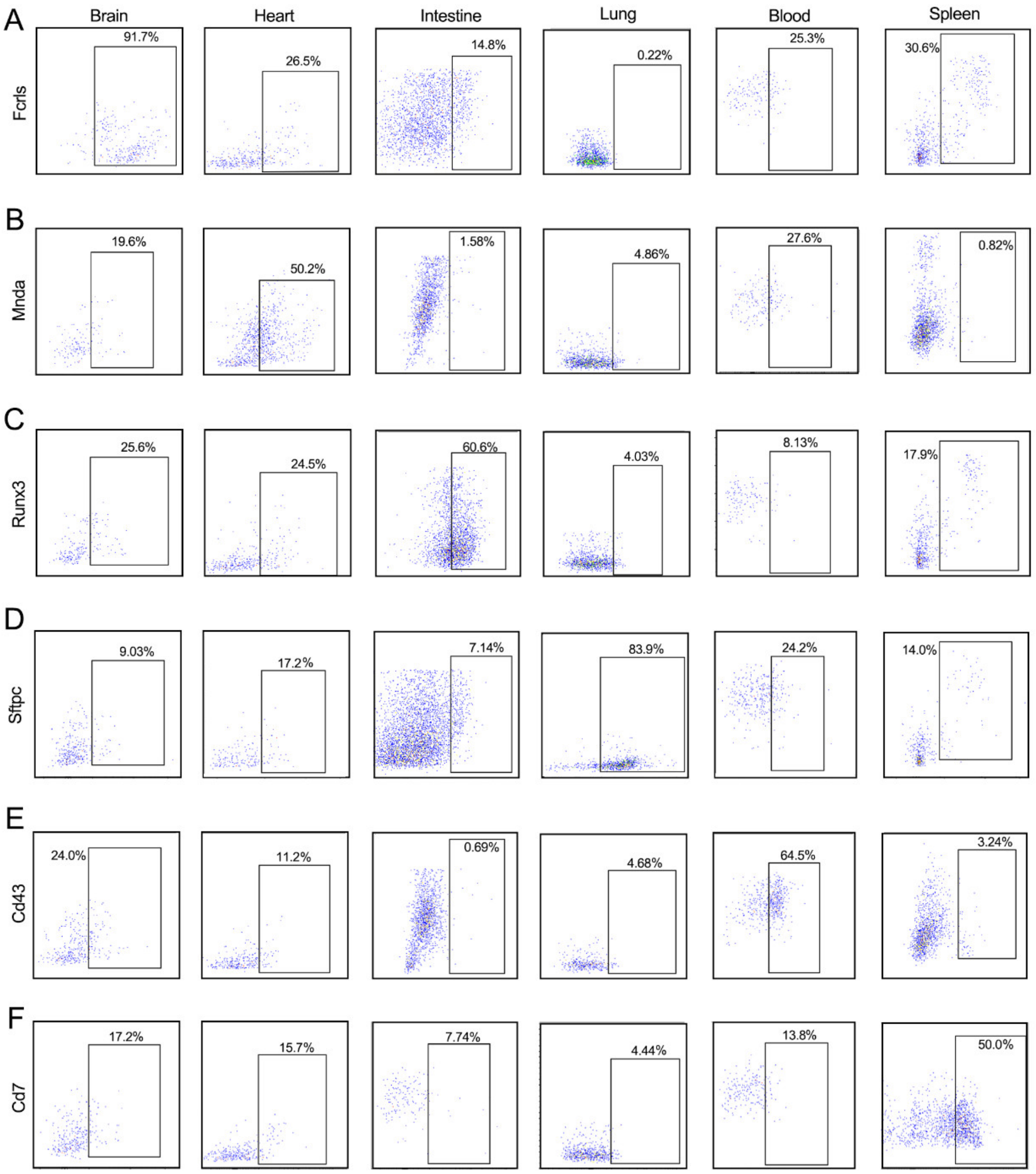

Figure S3. The marker genes of PBMCs and different tissue-resident macrophages. The proportion of Fcrls$^+$ (A), Mnda$^+$ (B), Runx3$^+$ (C), Sftpc$^+$ (D), Cd43$^+$ (E) and Cd7$^+$ (F) cells in pbmc or resident macrophages of brain, heart, intestine, lung and spleen

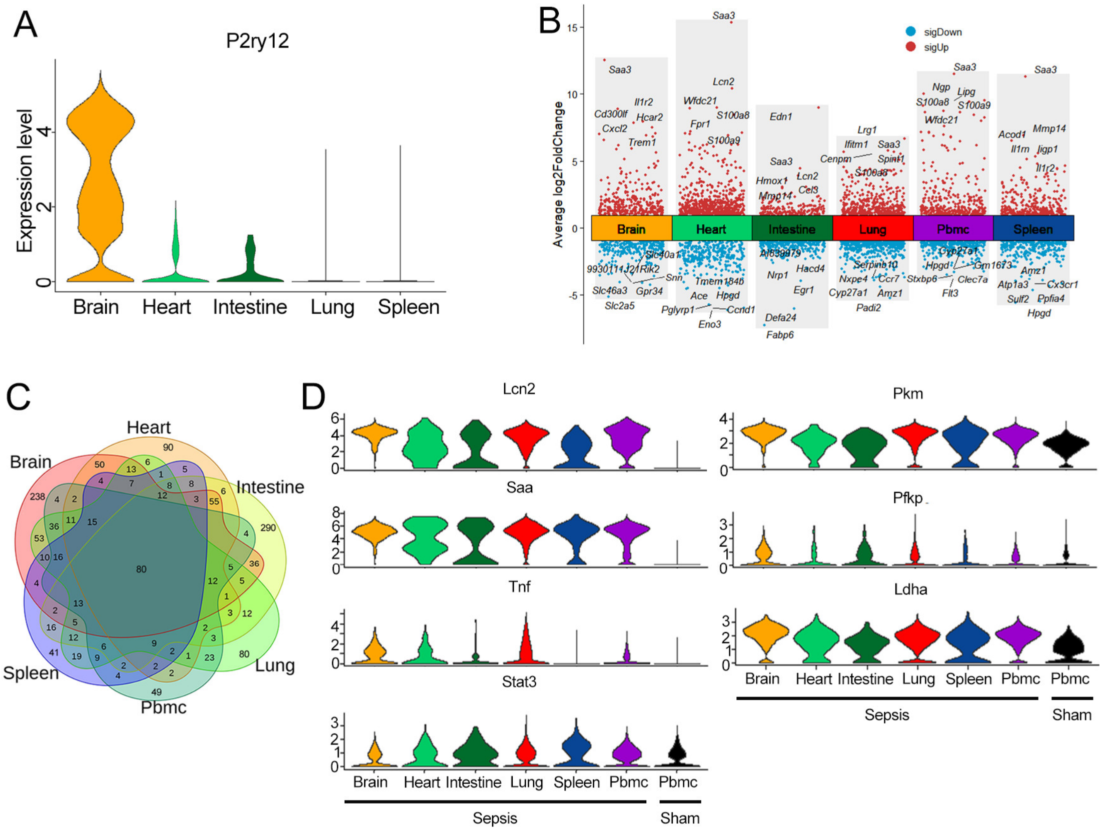

Figure S4. (A) The expression of *P2ry12* in resident macrophages of brain, heart, intestine, lung and spleen. (B) The differentially expressed genes of exogenous monocytes in different tissues after sepsis. (C) Venn plot shows the up-regulated genes of exogenous monocytes. (D) The expression of *Lcn2*, *Saa3*, *Tnf*, *Stat3*, *Pkm*, *Pfkp* and *Ldha* in exogenous monocytes and PBMCs.

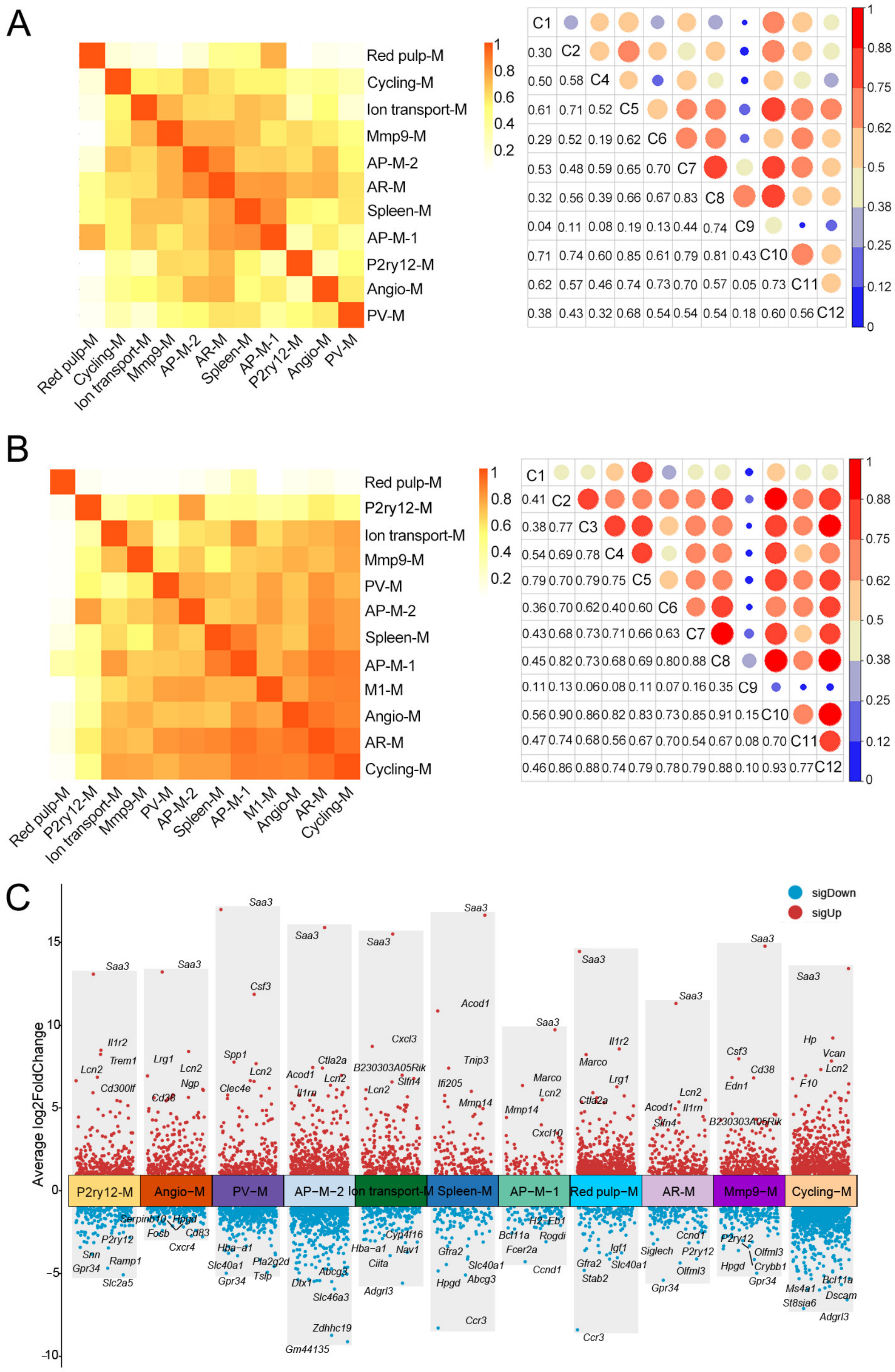

Figure S5. The changes of functional subtypes of resident macrophages after sepsis. (A) Pearson correlation between functional subtypes under steady state. (B) Pearson correlation between functional subtypes after sepsis. (C) The differentially expressed genes of functional subtypes after sepsis.

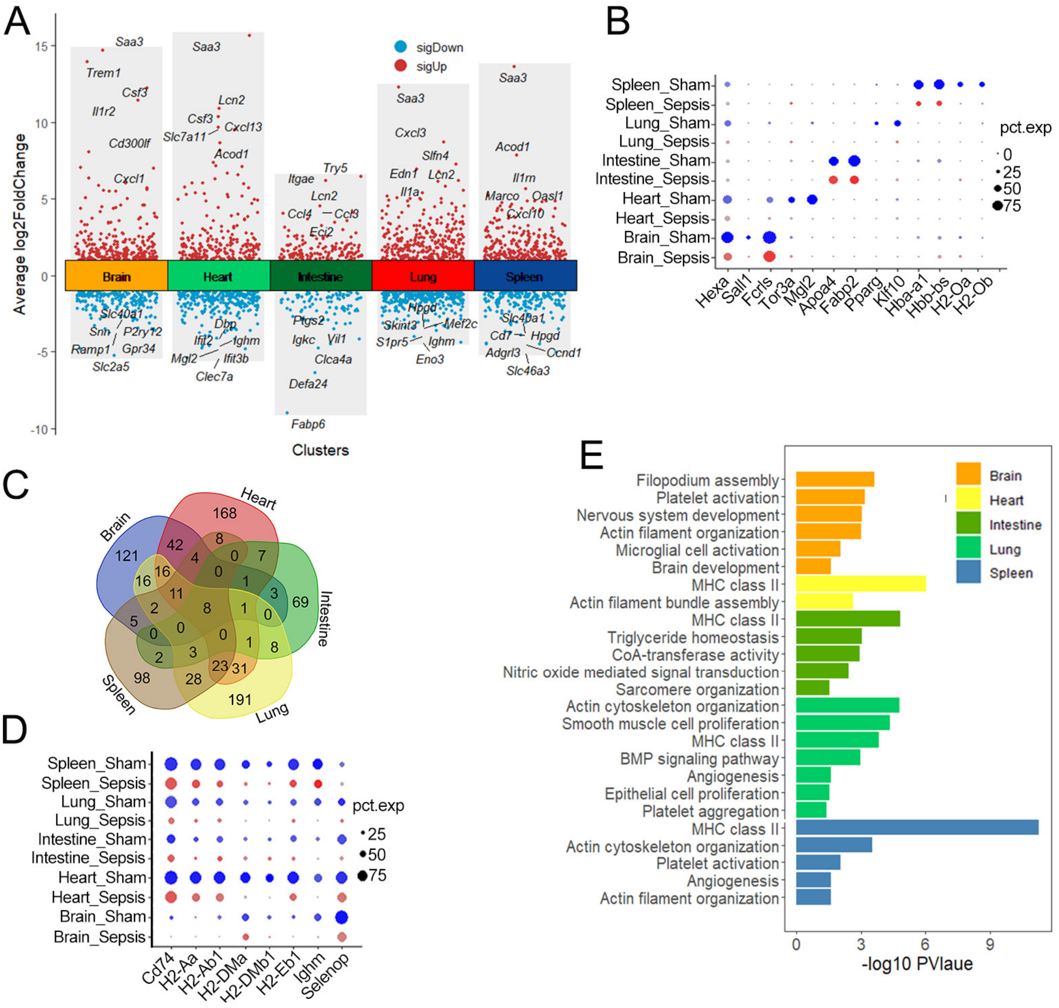

Figure S6. The differentially expressed genes of tissue-resident macrophages after sepsis. (A) The differentially expressed genes of resident macrophages in each tissue after sepsis. (B) Tissue-specifically downregulated genes. (C) Venn plot shows the down-regulated in different tissues. (D) 8 genes consistently down-regulated in different tissues after sepsis. (E) Functional enrichment analysis of the down-regulated genes in resident macrophages of each tissue.

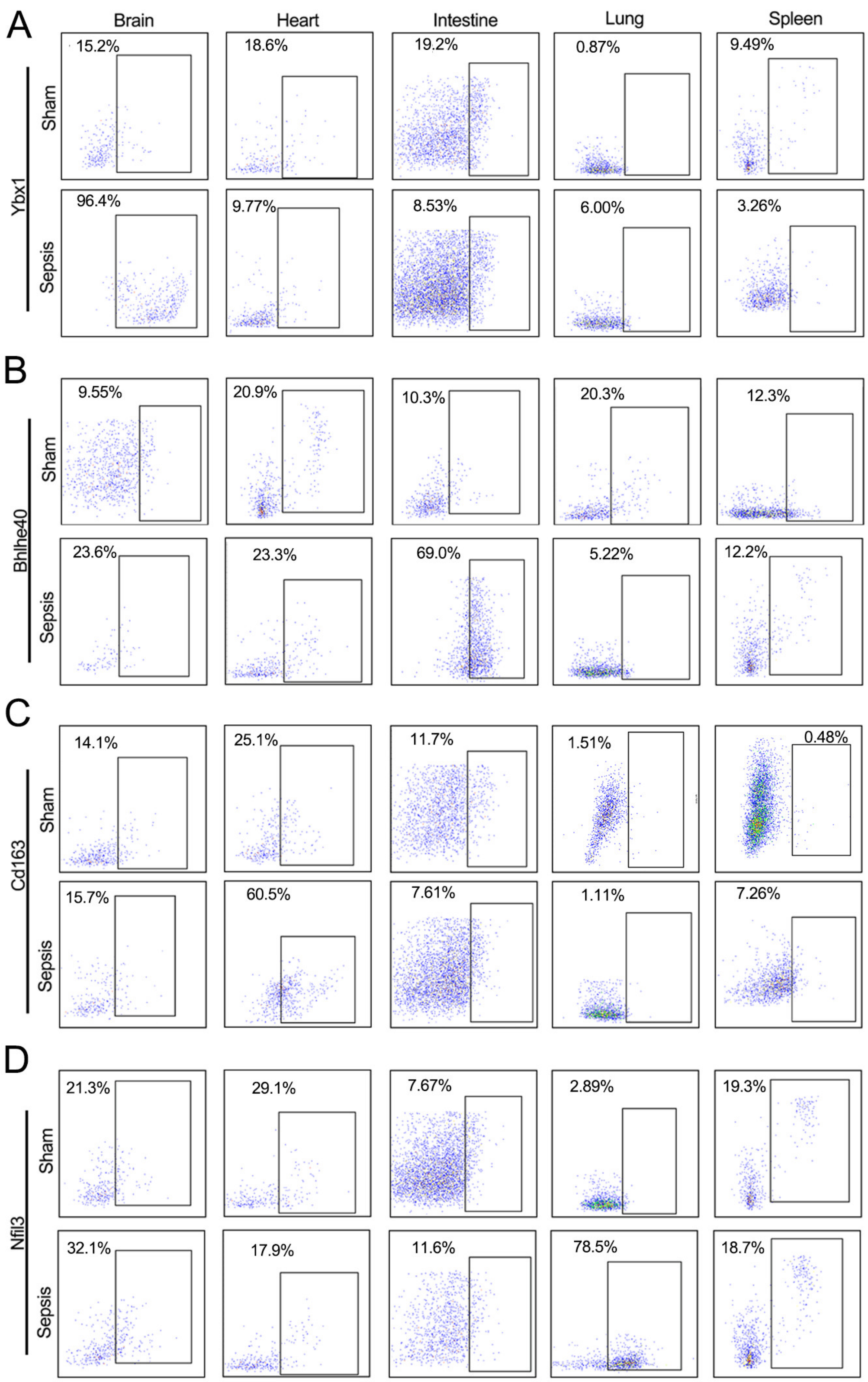

Figure S7. Flow cytometry revealed the proportions of $Ybx1^{+}$ (A), $Bhlhe40^{+}$ (B), $Cd163^{+}$ (C) and $Nfil3^{+}$ (D) cells in resident macrophages of different tissues from mice with sham and sepsis.

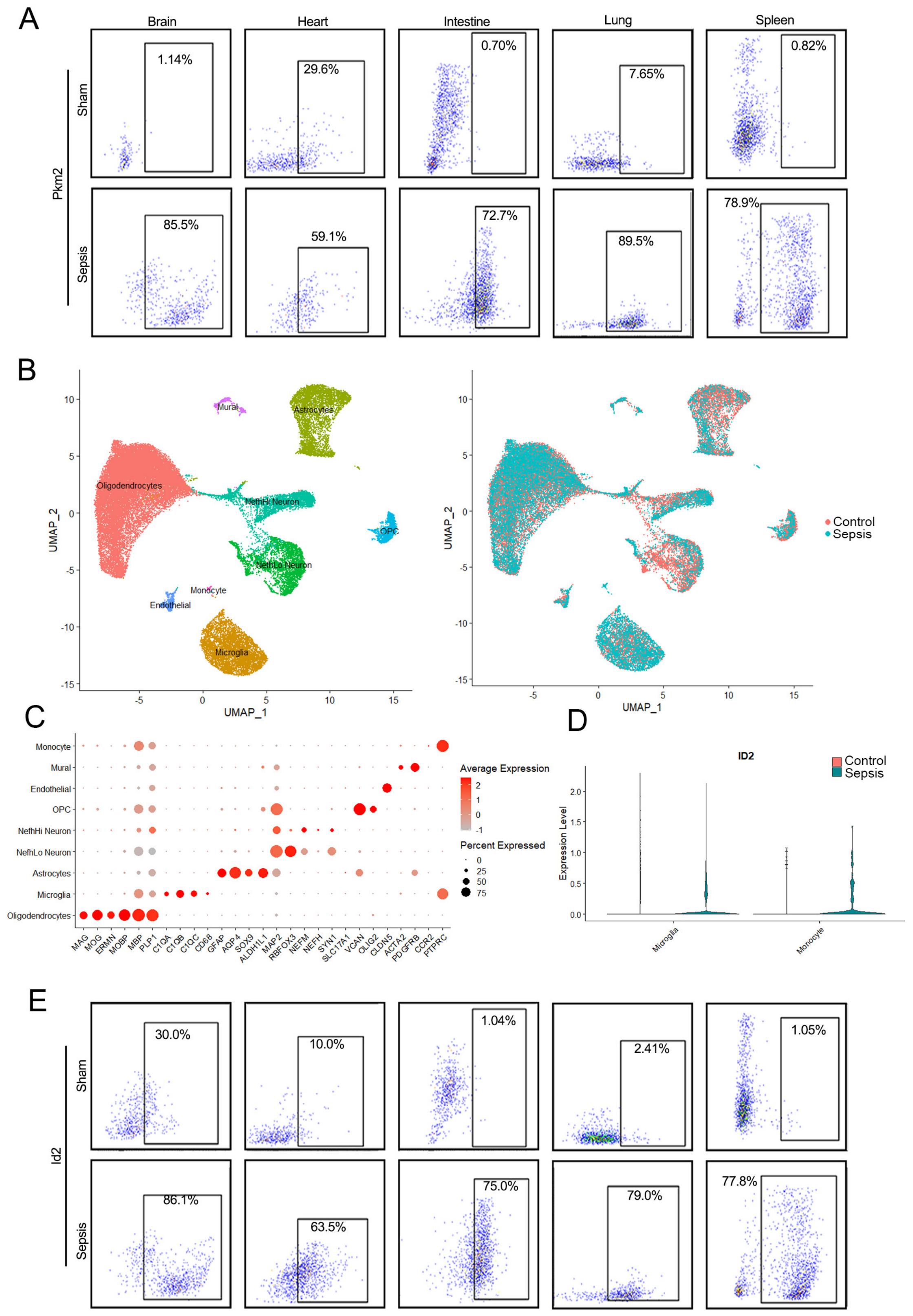

Figure S8. (A) Flow cytometry revealed the proportions of Pkm2^{+} cells in resident macrophages of different tissues from mice with sham and sepsis. (B) UMAP plots show the cell types of hippocampus from septic patients and age-matched controls. (C) DotPlot show the expression of marker genes in each cell type. (D) The expression of

ID2 in microglia and monocytes. (E) Flow cytometry revealed the proportions of Id2^{+} cells in resident macrophages of different tissues from mice with sham and sepsis.

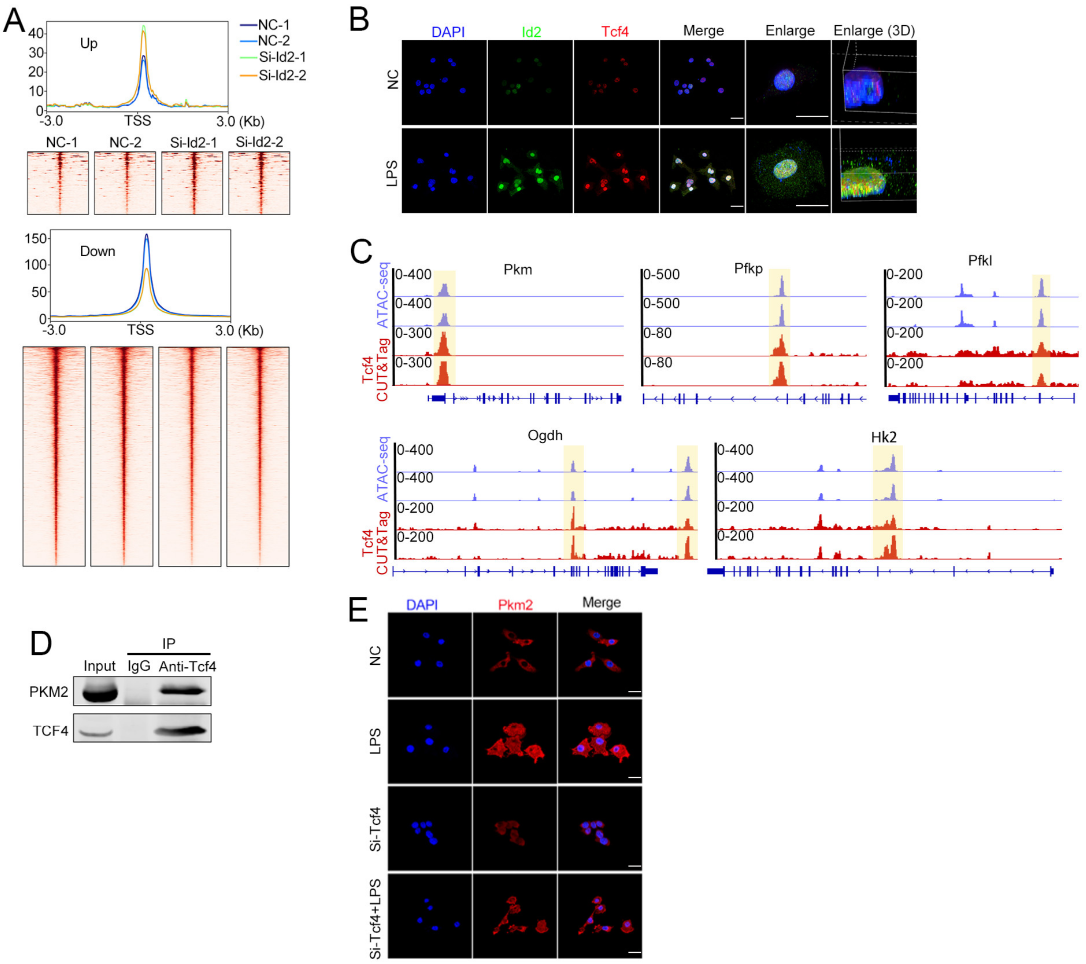

Figure S9. *Id2* cooperates with *Tcf4* to promote chromatin accessibility of *Pkm* and regulate its expression. (A) Peaks with increased (up) and decreased (down) chromatin accessibility. (B) Representative images illustrating Id2 (green) and Tcf4 (red) colocalize in RAW264.7 cells with DAPI-stained nuclei (blue) under normal control and 1 µg/mL LPS-treated. Each dot represents one experiment, scale bar, 10µm, n =3. (C) Integrative Genomics Viewer tracks display ATAC-seq reads and Anti-Tcf4 CUT&Tag reads along the indicated genes. (D) Coimmunoprecipitation assay of the Tcf4-Pkm2 interaction in RAW264.7 cells. (E) Representative images illustrating the expression of Pkm2 after siRNA-mediated knockdown of Tcf4. Each dot represents one experiment, scale bar, 10µm, n =3.
